# Development and evaluation of a culture-free microbiota profiling platform (MYcrobiota) for clinical diagnostics

**DOI:** 10.1007/s10096-018-3220-z

**Published:** 2018-03-16

**Authors:** Stefan A. Boers, Saskia D. Hiltemann, Andrew P. Stubbs, Ruud Jansen, John P. Hays

**Affiliations:** 1 Department of Medical Microbiology and Infectious Diseases, Erasmus University Medical Centre Rotterdam (Erasmus MC), Wytemaweg 80, 3015 CN Rotterdam, the Netherlands; 2 Department of Bioinformatics, Erasmus University Medical Centre Rotterdam (Erasmus MC), Rotterdam, the Netherlands; 3 Department of Molecular Biology, Regional Laboratory of Public Health Kennemerland, Haarlem, the Netherlands

**Keywords:** Microbiota, MYcrobiota, 16S rRNA gene sequencing, Clinical diagnostics, Micelle PCR, Bioinformatics pipeline

## Abstract

Microbiota profiling has the potential to greatly impact on routine clinical diagnostics by detecting DNA derived from live, fastidious, and dead bacterial cells present within clinical samples. Such results could potentially be used to benefit patients by influencing antibiotic prescribing practices or to generate new classical-based diagnostic methods, e.g., culture or PCR. However, technical flaws in 16S rRNA gene next-generation sequencing (NGS) protocols, together with the requirement for access to bioinformatics, currently hinder the introduction of microbiota analysis into clinical diagnostics. Here, we report on the development and evaluation of an “end-to-end” microbiota profiling platform (MYcrobiota), which combines our previously validated micelle PCR/NGS (micPCR/NGS) methodology with an easy-to-use, dedicated bioinformatics pipeline. The newly designed bioinformatics pipeline processes micPCR/NGS data automatically and summarizes the results in interactive, but simple web reports. In order to explore the utility of MYcrobiota in clinical diagnostics, 47 clinical samples (40 “damaged skin” samples and 7 synovial fluids) were investigated using routine bacterial culture as comparator. MYcrobiota confirmed the presence of bacterial DNA in 37/37 culture-positive samples and detected bacterial taxa in 2/10 culture-negative samples. Moreover, 36/38 potentially relevant aerobic bacterial taxa and 3/3 mixtures of anaerobic bacteria were identified using culture and MYcrobiota, with the sensitivity and specificity being 95%. Interestingly, the majority of the 448 bacterial taxa identified using MYcrobiota were not identified using culture, which could potentially have an impact on clinical decision-making. Taken together, the development of MYcrobiota is a promising step towards the introduction of microbiota analysis into clinical diagnostic laboratories.

## Introduction

The detection, identification, and further characterization of pathogenic microorganisms are the major step in establishing appropriate (antibiotic) treatment for infectious diseases. However, the causative microorganism of an infection may not always be detected using current “gold standard” culturing techniques. Further, most molecular-based detection methods, e.g., PCR, require a priori knowledge of the potential pathogen before a test is performed. To overcome these limitations, the bacterial composition can be defined and genera identified using a culture-free, broad-range PCR strategy that targets the prokaryotic 16S rRNA gene followed by next-generation sequencing (NGS) [1]. However, to date, 16S rRNA gene NGS methods to profile microbial compositions have been focused on research questions mostly, with only a few studies having evaluated the utility of 16S rRNA gene NGS methods for clinical microbiology [2, 3]. Currently, the utilization of 16S rRNA gene NGS methods within routine clinical diagnostics has been hindered by issues relating to the generation of PCR artifacts (e.g., chimera formation and PCR competition) and the susceptibility of 16S rRNA gene NGS methods to DNA contamination that is derived from the laboratory environment and/or the reagents/consumables used. These limitations hinder the standardization of current 16S rRNA gene NGS methods to such an extent that non-identical microbiota results may be obtained when repeatedly analyzing the same sample [4].

Recently, the authors published a micelle PCR/NGS (micPCR/NGS) methodology that limits the formation of chimeric sequences and prevents PCR competition via the clonal amplification of targeted 16S rRNA gene molecules [5]. In addition, the micPCR/NGS methodology allows for the utilization of an internal calibrator (IC) to calculate the number of 16S rRNA gene copies for each individual operational taxonomic unit (OTU) present within a (clinical) sample, which conveniently enables the subtraction of contaminating bacterial DNA via the quantification of 16S rRNA gene copies within negative extraction control (NEC) samples. The authors showed that the microbiota results obtained using micPCR/NGS possess a much higher accuracy (precision and trueness) compared to those obtained using traditional 16S rRNA gene NGS protocols and that the ability to determine and subtract contaminating 16S rRNA gene copies, results in contamination-free quantitative microbiota profiles—with a limit of detection (LOD) of only 25 16S rRNA gene copies per OTU [6]. This low LOD allows for the detection of bacterial OTUs at very low abundances or can confirm the absence of 16S rRNA gene copies in culture-negative results. Based on these findings, the authors suggested that the micPCR/NGS protocol could possess distinct advantages when processing clinical samples for microbiota profiling compared to traditional (semi-quantitative) 16S rRNA gene NGS methods that remain vulnerable to false-positive results (e.g., chimeric sequences or contaminant DNA) and inaccurate measurements of the OTU relative abundances in polymicrobial clinical samples due to template-specific variations in PCR efficiencies (i.e., PCR competition). However, the analysis of 16S rRNA gene NGS data depends on the use of bioinformatics tools that are complex for non-bioinformatics educated technicians/clinicians to utilize, and the required bioinformatics skills are nowadays mostly absent in clinical diagnostic laboratories.

In this publication, we designed an easy-to-use bioinformatics pipeline to determine bacterial taxa from 16S rRNA gene sequences that together with the micPCR/NGS strategy are part of an “end-to-end” microbiota profiling platform (MYcrobiota). The bioinformatics pipeline enables the full analyses of the NGS data obtained, from raw sequence files to final web reports that summarize the quantitative microbiota results, without the knowledge of command-line scripts that would normally be required by 16S rRNA gene NGS users. As a proof of principle, we explored the utility of MYcrobiota for use in the clinical diagnostic laboratory by processing a total of 47 clinical samples and then comparing the results to conventional “gold standard” culture results. The samples tested included 40 specimens that were obtained from a variety of damaged skin conditions for which a polymicrobial biomass was expected, and an additional 7 specimens, obtained from patients who were suspected of having (prosthetic) joint infections, for which a low bacterial biomass was expected.

## Materials and methods

### Ethics statement

An acknowledged national ethics committee from the Netherlands (Medisch Ethische Toetsingscommissie Noord-Holland, http://www.metc.nl) approved the study protocol (M015–021), and all experiments were performed on leftover material of the included clinical samples in accordance with the relevant guidelines and regulations. The national ethics committee waived the need for participant consent as all data were anonymized and analyzed retrospectively under code.

### Sample collection and study design

This study was performed retrospectively using 47 clinical samples obtained from 47 subjects. The results obtained by routine bacterial culturing methods had been used to guide patient treatment and care. In this study, we re-analyzed these samples using MYcrobiota and compared the results to the initial outcome of the culture results. The 47 samples included in this study were derived from wounds (22), ulcers (10), abscesses (5), puss (1), erysipelas (1), erythema (1), and 7 synovial fluids obtained from patients with suspected (prosthetic) joint infections.

### Routine bacterial culture

All samples were cultured according to standard laboratory protocols performed in our laboratory and stored at − 80 °C for subsequent MYcrobiota analysis. The routine bacterial culture methods included a 48-h incubation at 35 °C on tryptic soy agar plates with 5% sheep blood (TSASB, Oxoid), colistin aztreonam blood agar plates (CAP, Oxoid), and cystine lactose electrolyte deficient agar plates (CLED, Oxoid) under aerobic conditions; a 48-h incubation at 35 °C on chocolate agar with Vitox supplement (CHOCV, Oxoid) under 5% CO_2_ conditions; and a 48-h incubation at 35 °C on TSASB under anaerobic conditions. All Gram-negative rods, beta-hemolytic streptococci, *Staphylococcus aureus*, *Staphylococcus lugdunensis*, and anaerobic bacteria cultured were reported as potentially relevant bacteria, of which the identification of aerobic bacteria was obtained using MALDI-TOF mass spectrometry (Bruker). Note that in this study, we did not focus on optimizing culturing methods to increase the sensitivity of the culture results and the routine bacterial culture methods used may not be 100% efficient for culturing the bacteria that were detected with MYcrobiota.

### Micelle PCR and NGS

DNA was extracted from all 47 samples using the High Pure PCR Template Preparation Kit (Roche) according to the manufacturer’s instructions. In addition, DNA from the accompanying elution buffer was extracted as a NEC at the same time in order to allow the subtraction of contaminating bacterial DNA after NGS processing. The total number of 16S rRNA gene copies within each DNA extract was measured using a 16S rRNA gene quantitative PCR (qPCR) according to Yang et al. [7], after which each DNA extract was normalized to contain either 10,000, or < 1000 16S rRNA gene copies per microliter. A synthetic microbial community (SMC) sample, containing 10,000 16S rRNA gene copies of *Moraxella catarrhalis* (ATCC 25240), *Staphylococcus aureus* (ATCC 43300), *Haemophilus influenzae* (ATCC 10211), and *Clostridium perfringens* (ATCC 12915), was processed with each batch of clinical samples as a positive control (PC) sample. Prior to amplification by micPCR, 1000 or 100 16S rRNA gene copies of *Synechococcus* DNA were added respectively as IC to the normalized DNA extracts containing 10,000 or < 1000 16S rRNA gene copies per microliter. One hundred 16S rRNA gene copies of *Synechococcus* DNA were also added to the NEC DNA extract. The IC was used to express the resulting OTUs as a measure of 16S rRNA gene copies by the use of a correction factor (sample OTU copies = sample OTU reads × (initial IC copies/IC OTU reads)) as previously validated elsewhere [6].

16S rRNA gene amplicon library preparation using micPCR was performed as previously published [6], but we utilized a different micPCR primer set that made it possible to replace the former Roche 454 NGS platform with the Illumina MiniSeq platform. In this study, micPCR amplification was performed using modified 515F (5′-TCG TCG GCA GCG TCA GAT GTG TAT AAG AGA CAG TGY CAG CMG CCG CGG TAA-3′) and 806R (5′-GTC TCG TGG GCT CGG AGA TGT GTA TAA GAG ACA GGA CTA CNV GGG TWT CTA AT-3′) primers that amplified the V4 regions of 16S rRNA genes as recommended for Illumina NGS and which incorporated universal sequence tails at their 5′ ends to allow for a two-step amplification strategy. During the second round of amplification, dual indices and Illumina sequencing adapters were attached using the Nextera XT Index kit (Illumina). Paired-end sequencing of the 16S rRNA gene amplicon library was performed using the MiniSeq system in combination with the 2 × 150 bp MiniSeq System High-Output Kit (Illumina), after which FASTQ-formatted sequences were extracted from the MiniSeq machine for downstream analysis. We utilized the micPCR/NGS approach to process all samples, including the NEC and the PC, in triplicate in order to increase accuracy and to correct for contaminating bacteria DNA derived from the laboratory environment as previously described [6].

### Bioinformatics pipeline

**Fig. 1:**
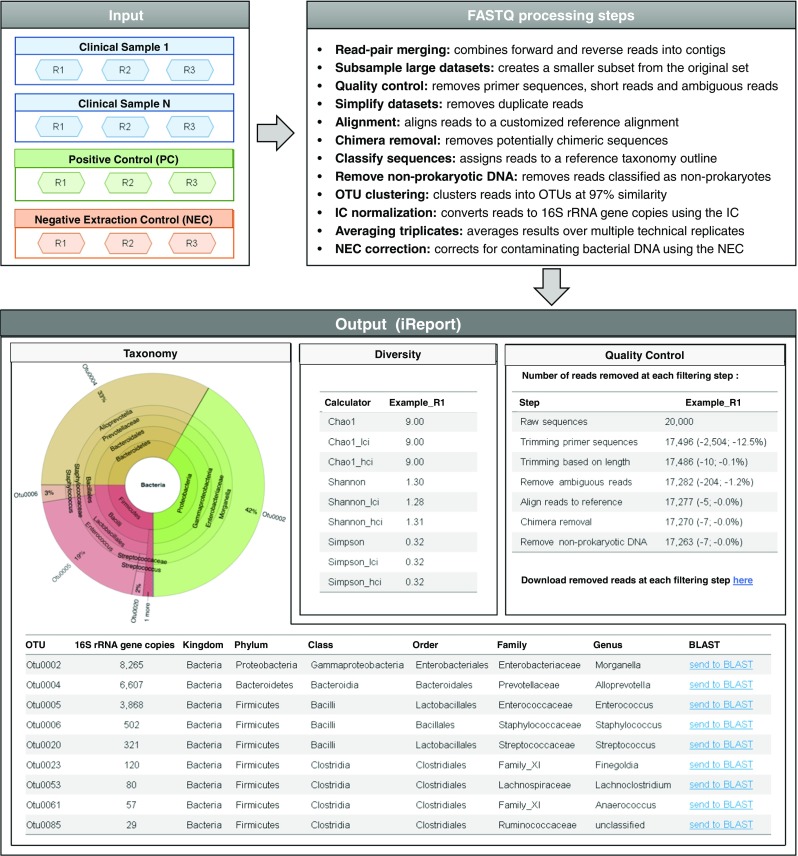
Schematical overview of the bioinformatics pipeline. FASTQ-formatted sequences obtained from triplicate experiments using micPCR/NGS (R1, R2, and R3) are automatically processed via the use of 32 (mothur) tools that have been integrated and combined in Galaxy as an “end-to-end” analysis service. The results obtained per sample (average of triplicate results) are presented to the user in a single, interactive iReport that consist of three tabs. The taxonomy tab visualizes and lists the resultant microbiota profiles. The diversity tab summarizes the results of three diversity calculators (Chao1, Shannon, and Simpson). The quality control tab provides an extensive overview of the quality control measurements during the analysis

The bioinformatics pipeline designed during this study consists of 23 well-established mothur tools (v.1.36) [8] and an additional 9 custom-made tools developed by the authors that have been integrated and combined in Galaxy as a full analysis service to deliver 16S rRNA gene analysis for micPCR/NGS experiments. Essentially, we have incorporated the functionality of mothur in Galaxy, which is a project dedicated to simplify the use of complex command-line bioinformatics tools (such as mothur) using a user-friendly web interface [9–11], and added new calculator tools to allow for a completely automatic processing of quantitative micPCR/NGS data. Importantly, the bioinformatics pipeline presents the microbiota results together with an extensive overview of the quality control measurements performed during the micPCR/NGS data analysis, to the user in an organized fashion via an interactive web report. The complete workflow of the bioinformatics pipeline is visualized in Fig. 1. All the tools required for the bioinformatics pipeline can be found in Galaxy’s Tool Shed (https://toolshed.g2.bx.psu.edu/). A workflow definition file can be downloaded from GitHub (https://github.com/ErasmusMC-Bioinformatics/MYcrobiota) and may be imported to any Galaxy platform, thereby offering the required set of bioinformatics tools. For more information on how to install and use this pipeline, please refer to the documentation in GitHub (https://github.com/ErasmusMC-Bioinformatics/MYcrobiota).

### Quantitative PCR methods

The total bacterial biomass within each DNA extract was measured using a 16S rRNA gene quantitative PCR (qPCR) that targets the 16S rRNA gene V5-V7 region, which is a different region of the 16S rRNA gene compared to MYcrobiota [7]. Therefore, the 16S rRNA gene qPCR is a complementary technique that enables the validation of the MYcrobiota process when determining the total number of 16S rRNA gene copies. For this, CT values were related to a serial dilution of the previous calibrated and normalized SMC sample that contained mixed and equimolar concentrations of four bacterial species and ranged from a total of 100 to 10,000 16S rRNA gene copies per PCR. In addition, the *S*. *aureus*-specific biomass was assessed within each DNA extract using a *S*. *aureus* qPCR that employs a *S*. *aureus*-specific marker as described by Martineau et al. [12]. Here, CT values were related to a serial dilution of only the calibrated *S*. *aureus* (ATCC 43300) DNA stock that ranged from a total of 10 to 10,000 copy numbers of the Martineau fragment. The PCRs were performed in 10-μL reaction volumes using the LightCycler 480 Probes Master (Roche) with the addition of 0.5 and 1.0 μM of each PCR primer for the 16S rRNA gene and *S*. *aureus* qPCRs respectively. Also, 0.25 μM of a Fam-labeled probe was added for the real-time detection of the 16S rRNA gene amplification, and 1× Resolight Dye (Roche) was added to the *S*. *aureus* qPCR in order to measure the *S*. *aureus* DNA amplification. All PCRs were performed using the following conditions: initial denaturation at 95 °C for 5 min followed by 45 cycles of PCR, with cycling conditions of 5 s at 95 °C, 10 s at 55 °C, and 30 s at 72 °C.

#### Availability of data and materials

The datasets generated and analyzed during the current study are available in the Sequence Read Archive repository with accession number SRP109023, https://www.ncbi.nlm.nih.gov/sra/?term=SRP109023.

## Results

### Development of an easy-to-use bioinformatics pipeline

In order to analyze 16S rRNA gene NGS data obtained using micPCR/NGS, we designed a Galaxy-based bioinformatics pipeline for use in clinical diagnostics. This workflow is largely based on the well-established standard operating procedure (SOP) defined by the creators of mothur [13]. We have adapted the SOP to our specific use-case by integrating several custom-made tools that allow for the subsampling of large datasets, the averaging over multiple technical replicates, converting the number of obtained sequence reads per OTU to 16S rRNA gene copies per OTU via the use of an IC, and correction for contaminating bacterial DNA via the use of NECs. All results are presented to the user as a single, interactive web report in Galaxy using the iReport tool [14]. The iReport was designed to visualize the resultant microbiota profiles using KRONA [15], list quantitative microbiota profiles in OTU tables (with the microbial load per OTU reported as 16S rRNA gene copies), summarize results of diversity calculators, and provide an extensive overview of the quality control measurements during the analysis. Importantly, the iReport is relatively small in size (~ 6 MB per sample for our datasets) that enables easy sharing and storage of 16S rRNA gene NGS results (Fig. 1).

### Validation of the MYcrobiota process

**Fig. 2:**
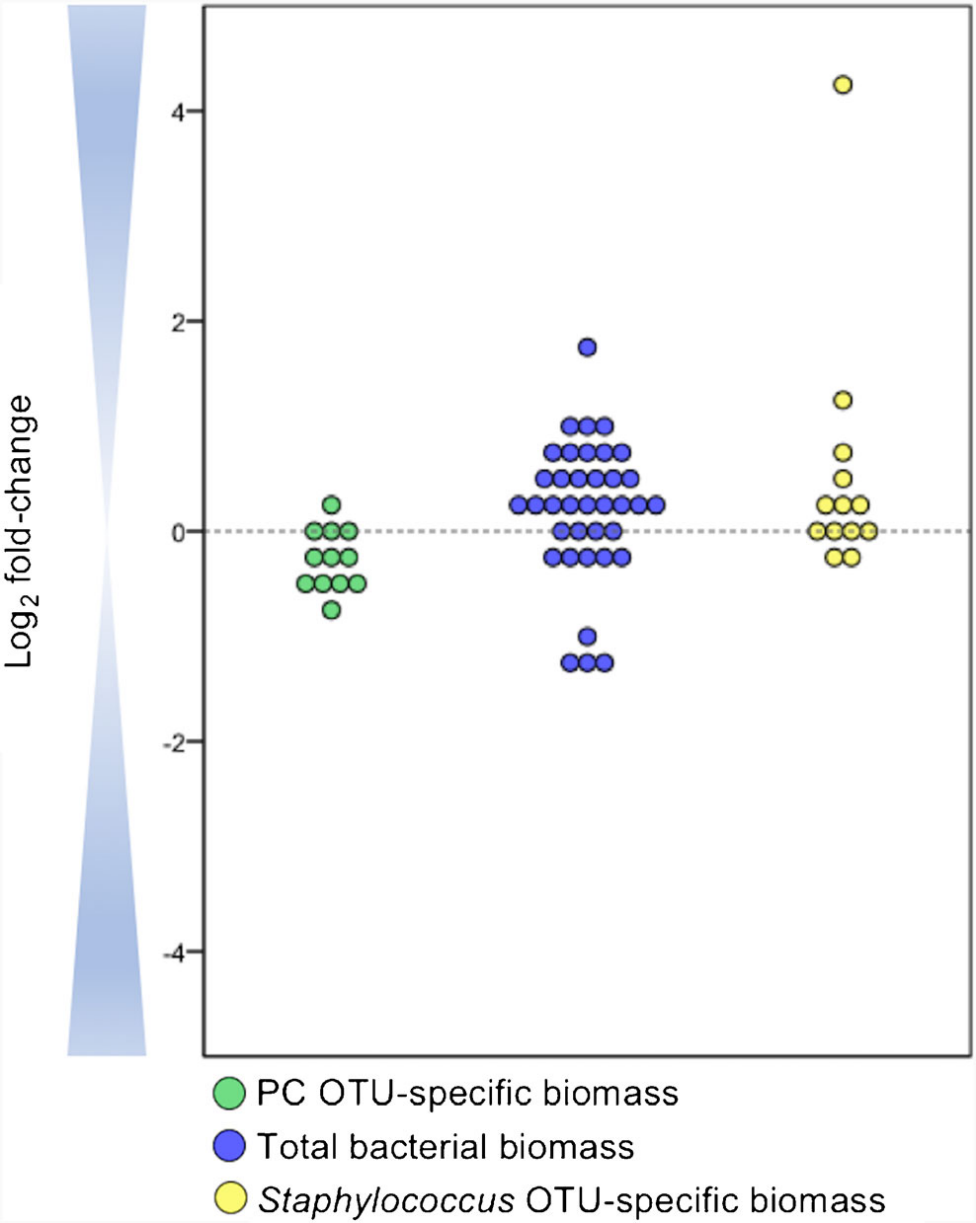
Accuracy of 16S rRNA gene copy determination using MYcrobiota. The expected number of 16S rRNA gene copies within the positive control (PC) was compared to the measured number of 16S rRNA gene copies using MYcrobiota (green dots). The PC contained 10,000 16S rRNA gene copies of four different bacterial species and was processed in three independent MYcrobiota experiments. The indirect estimation of the total bacterial biomass within 37 clinical samples using MYcrobiota was compared to the total 16S rRNA gene copies measured directly using a 16S rRNA gene qPCR (blue dots). The *Staphylococcus* OTU-specific biomass from 13 *S*. *aureus* culture-positive samples was compared to the *S*. *aureus* biomass detected directly using a *S*. *aureus*-specific qPCR (yellow dots). In order to compare the number of *S*. *aureus* genome copies estimated using qPCR to the number of 16S rRNA gene copies detected using MYcrobiota, the estimated *S*. *aureus* genome copies were first multiplied by a factor of 6 to correct for differences in copy numbers of the Martineau fragment and the 16S rRNA gene present on the *S*. *aureus* genome. The calculated differences between methods were plotted using a binary logarithmic scale

As shown in Fig. 2, MYcrobiota results obtained from the PC that was profiled in three independent experiments showed a median value of only a 1.3-fold (± 0.2) difference between the measured 16S rRNA gene copies per bacterial species and the expected 10,000 16S rRNA gene copies per bacterial species present in the PC. In addition, comparisons between the measured 16S rRNA gene copies determined in actual clinical samples using MYcrobiota compared to qPCR results revealed an average of only a 1.5-fold (± 0.5) and a 1.3-fold (± 0.4) difference for the total bacterial biomass and the *Staphylococcus* OTU-specific biomass respectively. Of note, 10 of the 47 clinical samples included in this study resulted in culture-negative results, and the absence of bacterial DNA in these samples was confirmed with both qPCR and MYcrobiota methods. Also, one discrepant sample was detected that showed a 20-fold higher abundance of staphylococci detected by MYcrobiota compared to that detected by qPCR. This result can be explained by the presence of *S*. *aureus* and *S.* non-*aureus* within this sample. In fact, the *S*. *aureus* qPCR showed a 100% specificity compared to *S*. *aureus* culture-positive results and indicates the presence of *S*. non-*aureus* bacteria within 7 additional samples in which the *Staphylococcus* OTU was detected using MYcrobiota but no *S*. *aureus* could be cultured. Taken together, these data demonstrate the accuracy of the MYcrobiota process and the ability to incorporate quantitative results obtained from additional (species-specific) qPCRs.

### Comparing MYcrobiota results to routine bacterial culture

In order to explore the utility of MYcrobiota in the field of clinical diagnostics, we processed a total of 47 clinical samples and compared the results to routine bacterial culture. All bacterial genera detected using culture and MYcrobiota are reported per sample in Table 1. Using standard bacterial culture techniques, our laboratory detected a total of 38 potentially relevant aerobic bacterial genera within 25 clinical samples and obtained a positive culture of a mixture of anaerobic bacteria in 3 samples. No bacteria were cultured from 10 samples, and an additional 10 samples resulted in the growth of bacteria that were all presumed to be commensal flora. In contrast, using MYcrobiota, we detected a total of 448 bacterial operational taxonomic units (OTUs) in 39 samples of which 337 OTUs (75%) could be identified as anaerobic bacterial genera that were detected in 21 samples. No bacterial DNA was measured in 8 out of 10 culture-negative samples. The sensitivity for bacterial culture detection by MYcrobiota was determined at 100% and the specificity at 83% using culture as “gold standard.”

**Table 1:**
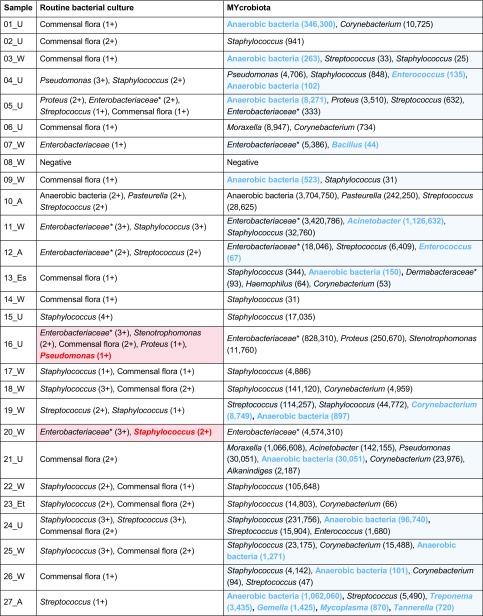

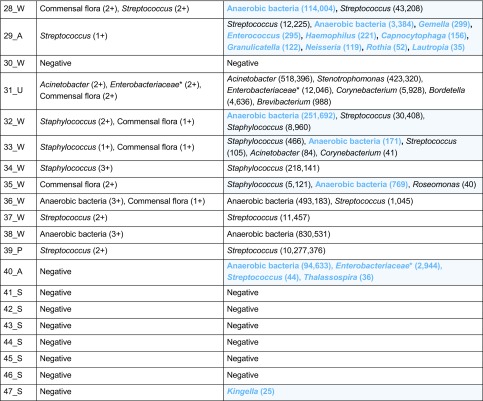
Bacterial genera identified from 47 clinical samples using routine bacterial culture and MYcrobiota

Samples were derived from wounds (W), ulcers (U), abscesses (A), puss (P), erysipelas (Es), erythema (Et), and suspected joint infections (S). Cultured bacteria other than Gram-negative rods, beta-hemolytic streptococci, *S*. *aureus*, *S*. *lugdunensis*, and anaerobic bacteria were reported as commensal flora. The semi-quantitative culture results are presented as 1+, 2+, 3+, or 4+, depending on which quadrants demonstrate bacterial growth. The presence of anaerobic bacteria was reported as either a positive or a negative result. Bacterial species and OTUs detected using culture and MYcrobiota respectively are grouped at the genus level to compare results. Red shades indicate bacterial genera that were only identified by culture and blue shades indicate bacterial genera that were only identified by MYcrobiota (with “commensal flora” culture results representing a positive detection signal for any kind of aerobic bacterial OTU identified by MYcrobiota). The number of 16S rRNA genes measured using MYcrobiota is indicated in parentheses

*Several bacterial genera that belong to the *Enterobacteriaceae* and *Dermabacteraceae* families could not be differentiated at a 97% similarity level using MYcrobiota

The majority of bacterial genera identified with culture were also identified using MYcrobiota. As shown in Table 2, MYcrobiota detected 36 of all 38 aerobic bacteria cultured on a genus-level taxonomy and confirmed the growth for anaerobic bacteria in 3 samples (sensitivity 95%; specificity 95%). Important to note, the two discrepant bacterial genera were measured using the micPCR/NGS strategy, but below the technique’s LOD of 25 16S rRNA gene copies per OTU. In contrast, the vast majority of bacterial genera identified with MYcrobiota were presumed to belong to the commensal flora using culture or were not cultured at all (Table 1). These additional taxa include potential pathogens such as the *Kingella* OTU that was detected from a synovial fluid sample obtained from a juvenile patient that was not detected using culture and was confirmed using a *Kingella kingae*-specific PCR.

**Table 2:** Comparison of the cultured bacterial taxa to MYcrobiota results

Bacterial taxa	Number of positive samples		Sensitivity (%)	Specificity (%)
	Routine bacterial culture	MYcrobiota		
*Acinetobacter*	1	4	100	98
Enterobacteriaceae*	7	8	100	98
*Pasteurella*	1	1	100	100
*Proteus*	2	2	100	100
*Pseudomonas*	2	2	67	100
*Staphylococcus*	14	20	93	100
*Stenotrophomonas*	1	2	100	100
*Streptococcus*	10	16	100	97
Anaerobic bacteria	3	21	100	71
Total	41	76	95	95

The culture results are restricted to genus-level classifications in order to compare the OTUs detected using MYcrobiota to the culture-based results. The presence of anaerobic bacteria was reported as either a positive or a negative result. “Commensal flora” culture results were interpreted as a positive detection signal for any kind of aerobic bacterial OTU identified by MYcrobiota to perform specificity calculations

*Several bacterial genera that belong to the *Enterobacteriaceae* family could not be differentiated at a 97% similarity level using MYcrobiota

## Discussion

In this study, we developed and explored the utility of an “end-to-end” microbiota profiling platform (MYcrobiota)—consisting of our previously published 16S rRNA gene sequencing methodology (micPCR/NGS) in combination with an easy-to-use bioinformatics pipeline—to investigate human samples for the clinical diagnostic laboratories. The bioinformatics pipeline designed during this study allows for a fully automated sequence interpretation of 16S rRNA gene NGS data that is obtained using the validated micPCR/NGS protocol without the need for advanced bioinformatics skills that are often unavailable in the clinical diagnostic laboratories. The MYcrobiota results are presented using (interactive) visualizations and tables, including an overview of all removed sequences during the analysis that allows for a manual evaluation of the quality measurements pre-installed within the bioinformatics pipeline. Moreover, connections of OTU representative sequences to the external NCBI database are available and can be used to ensure that the taxonomic identification of bacterial genera is correct [16]. Importantly, the summarizing reports are relatively small in size, and storage of these files enables the traceability of patient test results that are required for clinical diagnostic laboratories according to quality requirements.

Using MYcrobiota, we processed a total of 47 clinical samples and compared the results to routine bacterial culture. Our results showed that the majority of bacteria identified with culture were also identified with MYcrobiota, but the majority of bacterial taxa identified with MYcrobiota were not identified using culture. Many of the additional bacterial taxa identified using MYcrobiota are obligate anaerobes that were commonly detected as a large component of the microbial population in samples obtained from damaged skin sites, which is consistent with previous studies [17, 18]. Indeed, it is well known that anaerobic bacteria are able to cause serious and life-threatening infections but are often overlooked due to their requirement for appropriate methods of collection, transportation, and cultivation [19]. Therefore, the culture-free MYcrobiota detection platform can play an important role in the identification of the bacteriological etiology of anaerobic infections or any other infections caused by fastidious microorganisms. Of note, it could be argued that the development of extensive culture techniques (so-called culturomics) may eventually facilitate the successful culture of supposedly “non-culturable” microbial isolates [20].

In addition to the accurate detection and identification of bacterial OTUs within clinical samples, MYcrobiota also provides the relative abundances in combination with the absolute abundances for each detected bacterial OTU. This feature allows clinicians to obtain a comprehensive overview of the microbial composition of the clinical sample so that each quantified bacterial OTU, as well as the bacterial community as a whole, might be taken into account in clinical decision-making. Additionally, MYcrobiota allows for the removal of contaminating DNA from environmental sources in order to accurately and reliably investigate very low bacterial biomass, or no bacterial biomass, clinical samples [6]. For example, MYcrobiota confirmed the absence of 16S rRNA gene copies in eight of the ten samples that generated culture-negative results. The two discrepant samples contained either anaerobic bacteria or low amounts of the fastidious *Kingella* bacterium respectively. The ability to confirm culture-negative results improves the reliability of culture-negative diagnostic results. Additionally, the ability of MYcrobiota to detect bacterial OTUs at very low abundances makes MYcrobiota a suitable method to investigate normally sterile body sites, such as synovial fluids, cerebrospinal fluids, and blood samples. It should be noted however that the authors are aware of the fact that the construction of MYcrobiota is only a first step in the transition of microbiota research into actual clinical diagnostics. Extensive clinical and financial validation studies will be needed in order to validate and justify the routine introduction of molecular microbiota profiling methods into clinical diagnostic laboratories.

In conclusion, the stepwise development of MYcrobiota paves the way to introduce quantitative microbiota profiling into the clinical diagnostic laboratory. The method provides a highly accurate and comprehensive overview of the microbial composition of clinical samples or, alternatively, confirms the absence of 16S rRNA gene copies in culture-negative samples, using a standardized and validated 16S rRNA gene NGS workflow. Despite some shortcomings, e.g., lack of species identification and the inability to provide detailed information on antibiotic susceptibility, our data illustrates that MYcrobiota has promising applications in the field of clinical diagnostics and warrants investment in future studies to accurately evaluate the clinical relevance of 16S rRNA gene NGS results in clinical samples.
